# Regional Gray Matter Atrophy Coexistent with Occipital Periventricular White Matter Hyper Intensities

**DOI:** 10.3389/fnagi.2016.00214

**Published:** 2016-09-07

**Authors:** Dazhi Duan, Congyang Li, Lin Shen, Chun Cui, Tongsheng Shu, Jian Zheng

**Affiliations:** ^1^Department of Neurology, Xinqiao Hospital, Third Military Medical UniversityChongqing, China; ^2^Department of Neurology, Chengdu Military General HospitalChengdu, China; ^3^Department of Radiology, Xinqiao Hospital, Third Military Medical UniversityChongqing, China

**Keywords:** magnetic resonance imaging, occipital periventricular hyperintensities, voxel-based morphometry analysis, gray matter atrophy, LDL-C

## Abstract

White matter hyperintensities (WMHs) and brain atrophy often coexist in the elderly. Additionally, WMH is often observed as occipital periventricular hyperintensities (OPVHs) with low-grade periventricular (PV) white matter (WM) lesions and is usually confined within an anatomical structure. However, the effects of OPVHs on gray matter (GM) atrophy remain largely unknown. In this study, we investigated GM atrophy in OPVHs patients and explored the relationship between such atrophy and clinical risk factors. T1-weighted and T2-weighted Magnetic resonance imaging (MRI) were acquired, and voxel-based morphometry (VBM) analysis was applied. The clinical (demographic and cardiovascular) risk factors of the OPVHs patients and healthy controls were then compared. Lastly, scatter plots and correlation analysis were applied to explore the relationship between the MRI results and clinical risk factors in the OPVHs patients. OPVHs patients had significantly reduced GM in the right supramarginal gyrus, right angular gyrus, right middle temporal gyrus, right anterior cingulum and left insula compared to healthy controls. Additionally, OPVHs patients had GM atrophy in the left precentral gyrus and left insula cortex, and such atrophy is associated with a reduction in low-density lipoprotein cholesterol (LDL-C) and apolipoprotein-B (Apo-B).

## Introduction

White matter hyperintensities (WMHs) and brain atrophy often coexist in the elderly. Furthermore, WMHs can be divided into paraventricular WMHs (PVHs) and deep white matter hyperintensities (DWMHs; Katsumata et al., 2010). Previous studies have suggested that PVHs and DWMHs have different clinical and pathological features with PVHs related to brain atrophy and DWMHs related to cerebrovascular diseases (Barber et al., 2000). It should be noted that the degree of cognitive impairment associated with WMHs also depends on the volume and location of the lesions, as well as other factors such as brain reserve (Brickman et al., 2011; Murray et al., 2011; Smith et al., 2011; Hulst et al., 2013). Additionally, the impact of WMHs location is often assessed by dividing the brain into different regions, e.g., the occipital, temporal, frontal and parietal lobes, as well as the basal ganglia and infratentorial region. In particular, WMH is often observed as occipital periventricular hyperintensities (OPVHs) with low-grade periventricular (PV) WM lesions and is usually confined within an anatomical structure. However, the effects of OPVHs on gray matter (GM) atrophy remain largely unknown.

Magnetic resonance imaging (MRI) is a non-invasive medical imaging technique that can detect WMHs using T1-weighted and T2-weighted images. Voxel-based morphometry (VBM) analysis was recently developed to increase the sensitivity of MRI in the investigation of focal differences in brain anatomy using Statistical parametric mapping (SPM). In this study, we investigated GM atrophy in OPVHs patients using T1-weighted and T2-weighted MRI with VBM analysis. Additionally, the clinical (demographic and cardiovascular) risk factors of the OPVHs patients and healthy controls were compared. Lastly, we explored the relationship between the MRI results and clinical risk factors in OPVHs patients.

## Materials and Methods

### Subjects

Patients with OPVHs (*n* = 97) and healthy controls (*n* = 73) underwent MRI scanning between 2012 July and 2015 July in the Department of Neurology in Xinqiao Hospital, Third Military Medical University. The subjects (age range: 55–85 years old) were restricted from vitamin 12 intake for 3 months prior to scanning. It should be noted that subjects with other diseases leading to white matter (WM) lesions, such as multiple sclerosis, toxic cerebral WM lesions, progressive multifocal leukoencephalopathy, atrial fibrillation and thyroid disease, were excluded from the study. Additionally, cerebrovascular disease induced by heart and aorta embolism, atrial fibrillation, valvulopathy, endocarditis, myocardiopathy, left atrioventricular valve stenosis or ventricular aneurysm, vasculitis, familial high homocysteine, infectious diseases, anemia or malignant disease, intracranial tumors, any systemic diseases, receiving special treatment such as radiotherapy, drug chemotherapy and biological therapy, as well as immune system diseases such as connective tissue or autoimmunity diseases, were also excluded from the study. It should be noted that OPVHs patients have OPVHs ≥1 according to the Fazekas scale. Additionally, the patients did not have lesions in other locations, and the healthy controls (CN) had no WMHs in any location of the brain. This study was approved by the medical ethics committee of Xinqiao Hospital, Third Military Medical University, Chongqing, China. Full written informed consent for participation was obtained from each subject.

The gender (Gen), age, hypertension (Htn) and diabetes mellitus (DM) history, current smoking (CS) and current alcohol use (CAU) were obtained from the patient-administered questionnaire. Total cholesterol (TC), high-density lipoprotein cholesterol (HDL-C), low-density lipoprotein cholesterol (LDL-C), triglycerides (TG), uric acid (UA), apolipoprotein-A1 (Apo-A1) and apolipoprotein-B (Apo-B) were also measured.

### MRI Protocols

All MRI data were obtained using a 3.0 Tesla scanner (General Electric, Milwaukee, WI, USA) with a 12-channel head coil. Fast spin-echo (FSE) T2-weighted images and fluid attenuation inversion recovery (FLAIR) T1-weighted images were acquired with TE/TR = 112.2/3160 ms and TE/IT/TR = 27.072/860/1696.68 ms, respectively. All MRI images were acquired with a voxel size of 0.4688 × 0.4688 × 5 mm^3^, 20 sagittal slices and an in-plane resolution of 512 × 512. MRI images were then assessed visually by two neurologists using the Fazekas scale (Fazekas et al., 1987). Note that all subjects had no cerebral infarcts defined as focal hyperintensities in T2 images.

### Data Analysis

All statistical analyses of Demographic and clinical variables were performed using SPSS20 (IBMSPSS, Chicago, IL, USA). Data are presented as the means (standard deviation). Chi-square test and Student’s *t*-test were used to determine significant differences of the frequencies of categories and differences in continuous variables, respectively, between the groups.

The MRI data were processed using the SPM8 (Welcome Department of Imaging Neuroscience Group, London, UK^1^) with VBM implemented in the VBM8 toolbox^2^. Images were bias-corrected, tissue-classified and registered (Ashburner and Friston, 2005). Subsequently, VBM analyses were performed on the normalized GM and WM segments. Finally, the images were smoothed with a Gaussian kernel of 8 mm full width at half maximum (FWHM). Voxel-wise GM differences between OPVHs and the CN group were examined using the independent-sample *t*-test. To avoid possible edge effects between different tissue types, we excluded all voxels with GM values of less than 0.09 (absolute threshold masking). We also applied a threshold of *p* < 0.001 with 30-voxel clustering criteria and family-wise error rate (FEW) correction to eliminate potential type I errors. Age was used as a nuisance effect. Multiple regression VBM analysis was performed to explore the relationship between the MRI results and the clinical factors.

## Results

### Clinical Factors of the OPVHs Patients and CN

Table 1 summarizes the clinical factors of the OPVHs patients and CN. Chi-square test and Student’s *t*-test showed that TC, LDL-C and Apo-B were significantly lower in the OPVHs patients than the CN (*p* < 0.05). The OPVHs patients were significantly older than the CN. It should be noted that Gen, CS, DM, CAU and Htn showed no significant differences.

**Table 1:** **Differences in clinical risk factors between OPVHs patients and CN**.

Group	OPVHs	CN	*χ*^2^/t	*p*
*n*	97.00	73.00		
Gen (n, %male)^1^	61 (62.89)	35 (47.95)	3.78	0.05
Age (years)^2^	71.79 (8.29)	68.9 (7.91)	−4.87	0.00*
Fazekas score	2.73 (0.54)	0.0 (0.00)	\	\
CS (n, %)^1^	24 (24.74)	21 (28.77)	0.35	0.56
CAU (n, %)^1^	21 (21.65)	14 (19.18)	0.16	0.69
Htn (n, %)^1^	47 (48.45)	26 (35.62)	2.80	0.09
DM (n, %)^1^	18 (18.56)	8 (10.96)	1.86	0.17
TC^2^	4.61 (1.07)	4.81 (0.96)	2.05	0.04*
TG^2^	1.44 (0.76)	1.66 (1.17)	1.48	0.14
LDL_C^2^	2.51 (0.63)	2.83 (0.68)	3.20	0.00*
HDL_C^2^	1.34 (0.32)	1.29 (0.30)	−0.95	0.34
UA^2^	289 (75.30)	277.75 (67.33)	−1.01	0.31
Apo-A1^2^	1.30 (0.31)	1.25 (0.33)	−1.01	0.31
Apo-B^2^	0.84 (0.24)	0.91 (0.23)	2.24	0.03*

Data are presented as the means (standard deviation). Chi-square tests and Student’s t-test were used to determine significant differences in the frequencies of categories and differences in continuous variables, respectively, between the groups. TC, LDL-C and Apo-B were significantly lower in the OPVHs patients compared to the CN (p < 0.05). Regarding age, OPVHs patients were significantly older than the CN. It should be noted that the Gen, CS, DM, CAU, and Htn had no significant differences. Abbreviations are as follows: OPVHs, occipital periventricular hyperintensities; CN, healthy controls, Gen, gender; CS, current smoking; CAU, current alcohol use, Htn, hypertension; DM, diabetes mellitus; TC, total cholesterol; TG, triglyceride; LDL-C, low-density lipoprotein cholesterol; HDL-C, high-density lipoprotein cholesterol; UA, uric acid; Apo-A1, apolipoprotein-A1; and Apo-B, apolipoprotein-B; ^1, 2, *^denote chi-square test, Student’s t-test and p < 0.05, respectively.

### Comparison Between MRI Results of OPVHs Patients and CN

**Figure 1:**
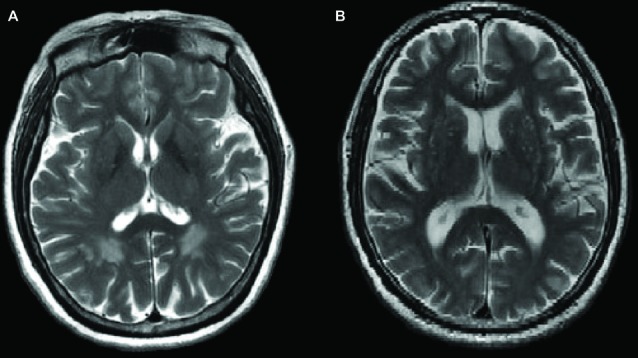
**Two typical T2-weighted Magnetic resonance imaging (MRI) images obtained from a occipital periventricular hyperintensities (OPVHs) patient (A) and a healthy control (CN) (B).** White matter hyperintensities (WMHs) were bright with higher signal intensities (A). Note that subjects had cerebral infarcts, defined as focal hyperintensities on T2 images.

**Figure 2:**
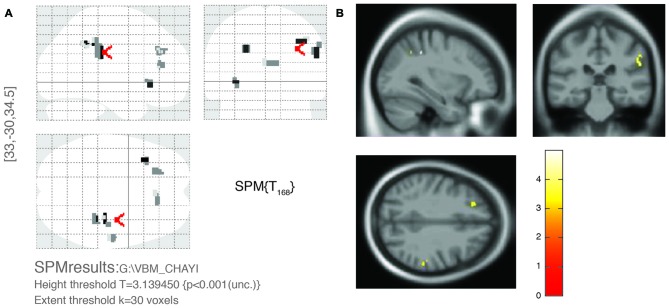
**Voxel-based morphometry (VBM) results of gray matter (GM) atrophy in OPVHs patients compared with CN. (A)** Statistical parametric mapping (SPM) results in various brain regions. **(B)** GM atrophy was found in the right supramarginal gyrus, right angular gyrus, right middle temporal gyrus, right anterior cingulum and left insula compared to the CN (*P* < 0.001, uncorrected, with 30-voxel clustering criteria).

Figure 1 shows the comparison between two typical T2-weighted MRI images obtained from a OPVHs patient and a CN. WMHs were bright with higher signal intensities. Note that no subjects had cerebral infarcts defined as focal hyperintensities on T2 images. Figure 2 and Table 2 show the VBM results: OPVHs patients had significantly reduced GM in the right supramarginal gyrus, right angular gyrus, right middle temporal gyrus, right anterior cingulum and left insula compared to the CN (*P* < 0.001, uncorrected, with 30-voxel clustering criteria).

**Table 2:** **VBM results of the gray matter atrophy in OPVHs patients compared with CN**.

Brain area	*T*	MNI coordinate		
		*x*	*y*	*z*
Right supramarginal gyrus	4.620	33.0	−39.0	40.5
Right angular gyrus	4.087	55.5	−31.5	30.0
Left insula	4.235	−36.0	21.0	−3.0
Right middle temporal lobe	4.260	39.0	−48.0	40.5
Right anterior cingulum	3.740	1.5	34.5	18.0
Left temporal lobe	3.525	−24.0	33.0	34.5

*Differences in brain regions were obtained by subtracting the results for the OPVHs patients from those of the CNs using the two independent samples t-test (p < 0.001, uncorrected, with a clustering criteria of voxels > 30). OPVHs patients had significantly reduced gray matter in the right supramarginal gyrus, right angular gyrus, right middle temporal gyrus, right anterior cingulum and left insula compared to the CN. Abbreviations are as follows: MNI coordinates, Montreal Neurological institute coordinates*.

### Multiple Regression VBM Analyses

**Figure 3:**
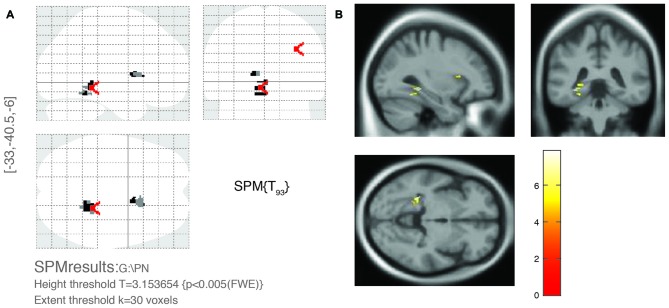
**Significant brain area of OPVHs in multivariate regression analyses. (A)** SPM results in various brain regions. **(B)** 3D brain map. GM atrophy was found in the left precentral gyrus and left insula cortex of patients with OPVHs in a multivariate regression analysis (*P* < 0.05, FWE-corrected, with 30-voxel clustering criteria).

**Figure 4:**
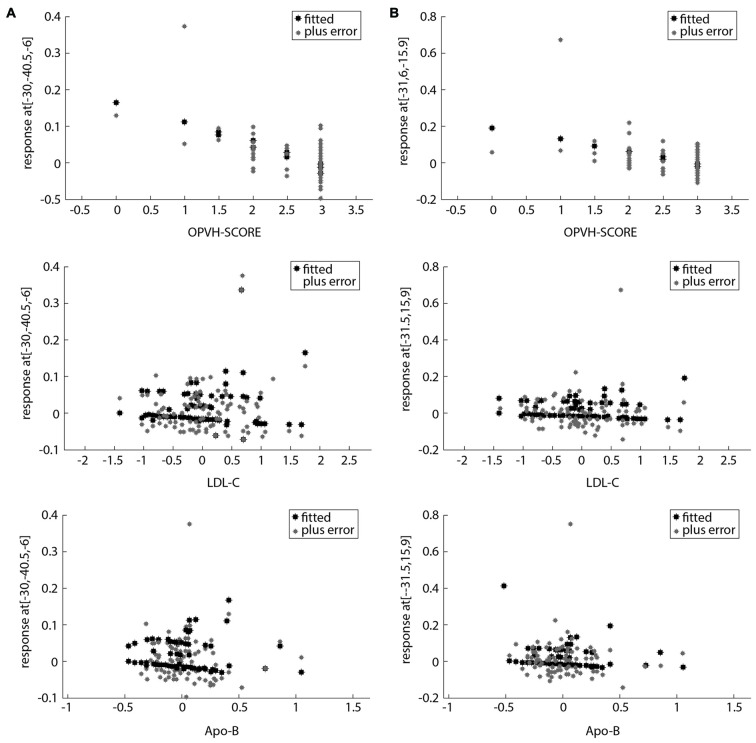
**Scatter plots of the relationship between potential clinical risk factors and the reduced GM in OPVHs patients. (A)** Scatter plots of the relationship between potential clinical risk factors and reduced GM in the left precentral gyrus.** (B)** Scatter plots of the relationship between potential clinical risk factors and reduced GM in left insula cortex. The reduced GM volume of the left precentral gyrus and insula cortex were correlated with Fazekas score, low-density lipoprotein cholesterol (LDL-C) and apolipoprotein-B (Apo-B).

For multivariate statistics, a *p* value of less than 0.05 in the univariate analysis was applied. Age and OPVHs as well as TC and LDL-C were strongly colinear in an orthogonal design; thus, only Fazekas score, LDL-C and Apo-B were applied for subsequent multivariate regression analyses. The reduced GM volumes of the left precentral gyrus and insula cortex were correlated with Fazekas score, LDL-C and Apo-B (*p* < 0.05, family-wise error (FSE)-corrected with 30-voxel clustering criteria; Table 3, Figures 3, 4).

**Table 3:** **Brain area of OPVHs with significance in multivariate regression analyses**.

Brain area	*T*	MNI coordinate		
		*x*	*y*	*z*
Left precentral gyrus	7.910	−30.000	−40.500	−6.000
Left insula cortex	7.630	−33.000	15.000	9.000

*Gray matter atrophy was found in the left precentral gyrus and insula cortex of patients with OPVHs in a multivariate regression analysis (p < 0.05, FWE-corrected, with 30-voxel clustering criteria)*.

## Discussion

OPVHs, a subgroup of WMHs, are frequently observed in elderly subjects (de Leeuw et al., 2001). Age and Htn have been reported to be the most common risk factors for WMHs (Liao et al., 1997; de Leeuw et al., 2002; Popa-Wagner et al., 2014, 2015; Pantoni et al., 2015), while hyperlipidemia, smoking, high body mass index, decreased vitamin B12 and alcohol may also be associated with WMHs (Breteler et al., 1994; Longstreth et al., 1996; Liao et al., 1997; Jeerakathil et al., 2004; Hickie et al., 2005; Murray et al., 2005; Stenset et al., 2006). Our study showed that Age, TC, LDL-C and Apo-B were statistically different between OPVHs group and CN group, OPVHs group had older age and lower TC, LDL-C and Apo-B compared with CN. Although the mechanism for the inverse correlation between TC, LDL-C and Apo-B and OPVHs is not fully understood, it should be noted that cholesterol may play an important role in neuron repair and remodeling in the central nervous system (Dietschy, 2009). Thus, the reduced cholesterol level may contribute to the reduction in WM.

Previous studies showed that WMHs have been associated with a number of adverse outcomes, including cognitive impairment, functional disability or even death (Scott et al., 2015; Yamawaki et al., 2015; Abraham et al., 2016). Additionally, the total volume and location of WMHs are important determinants of the clinical relevance (Swartz et al., 2003; Yoshita et al., 2006; Biesbroek et al., 2013). Visual and automated rating scales have been developed to assess the severity of WMHs (Fazekas et al., 1987; Scheltens et al., 1993; Gouw et al., 2006; Maillard et al., 2008; Maldjian et al., 2013). Two lesion subtypes have been proposed based on their proximity to the ventricles. These include PV lesions, which are contiguous with the ventricles, as well as DWMHs lesions, which are distinct and separate from the ventricles (Fazekas et al., 1993; Enzinger et al., 2006; Spilt et al., 2006; Rostrup et al., 2012). These lesion classifications have been supported by previous studies (Zhang et al., 2013). However, the cognitive impairment associated with WMHs may also depend on brain reserve (Brickman et al., 2011; Murray et al., 2011; Freret et al., 2015). WMHs and brain atrophy often coexist in the elderly (Aribisala et al., 2013). However, few studies have investigated GM atrophy in OPVHs patients. Our results demonstrate that GM atrophy occurred in the right supramarginal gyrus, right angular gyrus, right middle temporal gyrus, right anterior cingulum and left insula in OPVHs patients. These results are consistent with previous studies (Sepulcre et al., 2009; Raji et al., 2012).

Shared vascular risk factors may be an explanation for their concomitance. One explanation that has been proposed is that an impaired autoregulation due to the microangiopathy in combination with the luminal narrowing reduces the cerebral blood flow (Waldemar et al., 1994) and induce OPVHs. The hippocampus and amygdala are sensitive to hypoxia and ischemia (Pulsinelli et al., 1982; Cervós-Navarro and Diemer, 1991), which may lead to neuronal loss in these brain structures (Kril et al., 2002). However, disturbances of WM integrity could also be involved in the pathogenesis of brain atrophy. Our results showed that the left precentral gyrus and left insula cortex were correlated with Fazekas score, LDL-C and Apo-B. Notably, LDL-C and Apo-B are independently correlated with reduced voxels; however, their roles could not be determined. One possible mechanism is the loss of myelin, axons, and oligodendrocytes and other glial cells in the WM as a result of ischemic damage due to the underlying small-vessel disease (Pantoni and Garcia, 1997; von Bohlen und Halbach and Unsicker, 2002; Du et al., 2005). Another possibility is that WMHs influence GM with projecting tracts that modulate neurotransmitters (Bocti et al., 2005; Behl et al., 2007; Kim et al., 2012).

Our study was conducted using a cross-sectional design based in a hospital and had a relatively small sample size. A specifically designed, randomized, controlled prospective population-based study is warranted in the future. Future studies may also assess the role between OPVHs, LDL-C and Apo-B in GM atrophy in a potential mechanistic study.

## Conclusion

OPVHs patients had significantly reduced GM in the right supramarginal gyrus, right angular gyrus, right middle temporal gyrus, right anterior cingulum and left insula compared to healthy controls. Additionally, OPVHs patients had GM atrophy in the left precentral gyrus and left insula cortex, and such atrophy is associated with a reduction in LDL-C and Apo-B. OPVHs may lead to cerebral atrophy related to cognitive impairment. Future studies exploring the structural foundations of human cognitive impairment may benefit from our study.

## Author Contributions

The specific work of each author in this study was as follows: JZ: perception and design; final approval of the version to be published; DD: participation in the whole work; drafting of the article; data analysis; CL: demographic and cardiovascular risk factor data collection; LS: demographic and cardiovascular risk factor data collection; CC: MRI data acquisition and assessment; TS: MRI data acquisition and assessment.

## Conflict of Interest Statement

The authors declare that the research was conducted in the absence of any commercial or financial relationships that could be construed as a potential conflict of interest.

## Footnotes

^1^http://www.fil.ion.ucl.ac.uk/spm

^2^http://dbm.neuro.uni-jena.de/vbm.html

## References

[B1] Abraham H. M. Wolfson L. Moscufo N. Guttmann C. R. Kaplan R. F. White W. B. (2016). Cardiovascular risk factors and small vessel disease of the brain: blood pressure, white matter lesions and functional decline in older persons. J. Cereb. Blood Flow Metab. 36, 132–142. 10.1038/jcbfm.2015.121 26036933 PMC4758547

[B2] Aribisala B. S. Valdés Hernández M. C. Royle N. A. Morris Z. Muñoz Maniega S. Bastin M. E.. (2013). Brain atrophy associations with white matter lesions in the ageing brain: the Lothian Birth Cohort 1936. Eur. Radiol. 23, 1084–1092. 10.1007/s00330-012-2677-x 23114884

[B3] Ashburner J. Friston K. J. (2005). Unified segmentation. Neuroimage 26, 839–851. 10.1016/j.neuroimage.2005.02.018 15955494

[B4] Barber R. Gholkar A. Scheltens P. Ballard C. McKeith I. G. O’Brien J. T. (2000). MRI volumetric correlates of white matter lesions in dementia with Lewy bodies and Alzheimer’s disease. Int. J. Geriatr. Psychiatry 15, 911–916. 10.1002/1099-1166(200010)15:10<911::aid-gps217>3.0.co;2-t 11044873

[B5] Behl P. Bocti C. Swartz R. H. Gao F. Sahlas D. J. Lanctot K. L.. (2007). Strategic subcortical hyperintensities in cholinergic pathways and executive function decline in treated Alzheimer patients. Arch. Neurol. 64, 266–272. 10.1001/archneur.64.2.266 17296844

[B6] Biesbroek J. M. Kuijf H. J. van der Graaf Y. Vincken K. L. Postma A. Mali W. P.. (2013). Association between subcortical vascular lesion location and cognition: a voxel-based and tract-based lesion-symptom mapping study. The SMART-MR study. PLoS One 8:e60541. 10.1371/journal.pone.0060541 23593238 PMC3620525

[B7] Bocti C. Swartz R. H. Gao F. Q. Sahlas D. J. Behl P. Black S. E. (2005). A new visual rating scale to assess strategic white matter hyperintensities within cholinergic pathways in dementia. Stroke 36, 2126–2131. 10.1161/01.str.0000183615.07936.b6 16179569

[B8] Breteler M. M. van Swieten J. C. Bots M. L. Grobbee D. E. Claus J. J. van den Hout J. H.. (1994). Cerebral white matter lesions, vascular risk factors and cognitive function in a population-based study: the Rotterdam study. Neurology 44, 1246–1252. 10.1212/wnl.44.7.1246 8035924

[B9] Brickman A. M. Siedlecki K. L. Muraskin J. Manly J. J. Luchsinger J. A. Yeung L. K.. (2011). White matter hyperintensities and cognition: testing the reserve hypothesis. Neurobiol. Aging 32, 1588–1598. 10.1016/j.neurobiolaging.2009.10.013 19926168 PMC2891625

[B10] Cervós-Navarro J. Diemer N. H. (1991). Selective vulnerability in brain hypoxia. Crit. Rev. Neurobiol. 6, 149–182. 1773451

[B11] de Leeuw F. E. de Groot J. C. Achten E. Oudkerk M. Ramos L. M. Heijboer R.. (2001). Prevalence of cerebral white matter lesions in elderly people: a population based magnetic resonance imaging study. The Rotterdam Scan study. J. Neurol. Neurosurg. Psychiatry 70, 9–14. 10.1136/jnnp.70.1.9 11118240 PMC1763449

[B12] de Leeuw F. E. de Groot J. C. Oudkerk M. Witteman J. C. Hofman A. van Gijn J.. (2002). Hypertension and cerebral white matter lesions in a prospective cohort study. Brain 125, 765–772. 10.1093/brain/awf077 11912110

[B13] Dietschy J. M. (2009). Mutations in NPC1 and cholesterol metabolism in the brain. FASEB J. 23.

[B14] Du A. T. Schuff N. Chao L. L. Kornak J. Ezekiel F. Jagust W. J.. (2005). White matter lesions are associated with cortical atrophy more than entorhinal and hippocampal atrophy. Neurobiol. Aging 26, 553–559. 10.1016/j.neurobiolaging.2004.05.002 15653183

[B15] Enzinger C. Smith S. Fazekas F. Drevin G. Ropele S. Nichols T.. (2006). Lesion probability maps of white matter hyperintensities in elderly individuals: results of the Austrian stroke prevention study. J. Neurol. 253, 1064–1070. 10.1007/s00415-006-0164-5 16607471

[B16] Fazekas F. Chawluk J. B. Alavi A. Hurtig H. I. Zimmerman R. A. (1987). MR signal abnormalities at 1.5 T in Alzheimer’s dementia and normal aging. Am. J. Roentgenol. 149, 351–356. 10.2214/ajr.149.2.351 3496763

[B17] Fazekas F. Kleinert R. Offenbacher H. Schmidt R. Kleinert G. Payer F.. (1993). Pathologic correlates of incidental MRI white matter signal hyperintensities. Neurology 43, 1683–1689. 10.1212/wnl.43.9.1683 8414012

[B18] Freret T. Gaudreau P. Schumann-Bard P. Billard J. M. Popa-Wagner A. (2015). Mechanisms underlying the neuroprotective effect of brain reserve against late life depression. J. Neural Transm. (Vienna) 122, S55–S61. 10.1007/s00702-013-1154-2 24390152

[B19] Gouw A. A. Van der Flier W. M. van Straaten E. C. W. Barkhof F. Ferro J. M. Baezner H.. (2006). Simple versus complex assessment of white matter hyperintensities in relation to physical performance and cognition: the LADIS study. J. Neurol. 253, 1189–1196. 10.1007/s00415-006-0193-5 16998647

[B20] Hickie I. Naismith S. Ward P. B. Scott E. Mitchell P. Wilhelm K.. (2005). Vascular risk and low serum B12 predict white matter lesions in patients with major depression. J. Affect. Disord. 85, 327–332. 10.1016/j.jad.2004.11.003 15780703

[B21] Hulst H. E. Steenwijk M. D. Versteeg A. Pouwels P. J. Vrenken H. Uitdehaag B. M.. (2013). Cognitive impairment in MS: impact of white matter integrity, gray matter volume and lesions. Neurology 80, 1025–1032. 10.1212/wnl.0b013e31828726cc 23468546

[B22] Jeerakathil T. Wolf P. A. Beiser A. Massaro J. Seshadri S. D’Agostino R. B.. (2004). Stroke risk profile predicts white matter hyperintensity volume: the Framingham study. Stroke 35, 1857–1861. 10.1161/01.str.0000135226.53499.85 15218158

[B23] Katsumata T. Otori T. Nishiyama Y. Okubo S. Nishiyama Y. Nagayama H.. (2010). Correlation between insulin resistance and white matter lesions among non-diabetic patients with ischemic stroke. Neurol. Res. 32, 743–747. 10.1179/016164109x12608733393755 20223079

[B24] Kim S. H. Kang H. S. Kim H. J. Moon Y. Ryu H. J. Kim M. Y.. (2012). The effect of ischemic cholinergic damage on cognition in patients with subcortical vascular cognitive impairment. J. Geriatr. Psychiatry Neurol. 25, 122–127. 10.1177/0891988712445089 22689705

[B25] Kril J. J. Patel S. Harding A. J. Halliday G. M. (2002). Patients with vascular dementia due to microvascular pathology have significant hippocampal neuronal loss. J. Neurol. Neurosurg. Psychiatry 72, 747–751. 10.1136/jnnp.72.6.747 12023418 PMC1737900

[B26] Liao D. Cooper L. Cai J. Toole J. Bryan N. Burke G.. (1997). The prevalence and severity of white matter lesions, their relationship with age, ethnicity, gender and cardiovascular disease risk factors: the ARIC study. Neuroepidemiology 16, 149–162. 10.1159/000368814 9159770

[B27] Longstreth W. T. Jr. Manolio T. A. Arnold A. Burke G. L. Bryan N. Jungreis C. A.. (1996). Clinical correlates of white matter findings on cranial magnetic resonance imaging of 3301 elderly people. The Cardiovascular Health study. Stroke 27, 1274–1282. 10.1161/01.str.27.8.1274 8711786

[B28] Maillard P. Delcroix N. Crivello F. Dufouil C. Gicquel S. Joliot M.. (2008). An automated procedure for the assessment of white matter hyperintensities by multispectral (T1, T2, PD) MRI and an evaluation of its between-centre reproducibility based on two large community databases. Neuroradiology 50, 31–42. 10.1007/s00234-007-0312-3 17938898

[B29] Maldjian J. A. Whitlow C. T. Saha B. N. Kota G. Vandergriff C. Davenport E. M.. (2013). Automated white matter total lesion volume segmentation in diabetes. Am. J. Neuroradiol. 34, 2265–2270. 10.3174/ajnr.a3590 23868156 PMC4038900

[B31] Murray A. D. Staff R. T. McNeil C. J. Salarirad S. Ahearn T. S. Mustafa N.. (2011). The balance between cognitive reserve and brain imaging biomarkers of cerebrovascular and Alzheimer’s diseases. Brain 134, 3687–3696. 10.1093/brain/awr259 22102649

[B30] Murray A. D. Staff R. T. Shenkin S. D. Deary I. J. Starr J. M. Whalley L. J. (2005). Brain white matter hyperintensities: relative importance of vascular risk factors in nondemented elderly people. Radiology 237, 251–257. 10.1148/radiol.2371041496 16126931

[B32] Pantoni L. Fierini F. Poggesi A. LADIS Study Group. (2015). Impact of cerebral white matter changes on functionality in older adults: an overview of the LADIS study results and future directions. Geriatr. Gerontol. Int. 15, 10–16. 10.1111/ggi.12665 26671152

[B33] Pantoni L. Garcia J. H. (1997). Pathogenesis of leukoaraiosis: a review. Stroke 28, 652–659. 10.1161/01.str.28.3.652 9056627

[B34] Popa-Wagner A. Buga A. M. Popescu B. Muresanu D. (2015). Vascular cognitive impairment, dementia, aging and energy demand. A vicious cycle. J. Neural Transm. (Vienna) 122, S47–S54. 10.1007/s00702-013-1129-3 24337666

[B35] Popa-Wagner A. Buga A. M. Tica A. A. Albu C. V. (2014). Perfusion deficits, inflammation and aging precipitate depressive behaviour. Biogerontology 15, 439–448. 10.1007/s10522-014-9516-1 25033986

[B36] Pulsinelli W. A. Brierley J. B. Plum F. (1982). Temporal profile of neuronal damage in a model of transient forebrain ischemia. Ann. Neurol. 11, 491–498. 10.1002/ana.410110509 7103425

[B37] Raji C. A. Lopez O. L. Kuller L. H. Carmichael O. T. Longstreth W. T. Jr. Gach H. M.. (2012). White matter lesions and brain gray matter volume in cognitively normal elders. Neurobiol. Aging 33, 834.e7–834.e16. 10.1016/j.neurobiolaging.2011.08.010 21943959 PMC3248984

[B38] Rostrup E. Gouw A. A. Vrenken H. van Straaten E. C. Ropele S. Pantoni L.. (2012). The spatial distribution of age-related white matter changes as a function of vascular risk factors–results from the LADIS study. Neuroimage 60, 1597–1607. 10.1016/j.neuroimage.2012.01.106 22305990

[B39] Scheltens P. Barkhof F. Leys D. Pruvo J. P. Nauta J. J. Vermersch P.. (1993). A semiquantative rating scale for the assessment of signal hyperintensities on magnetic resonance imaging. J. Neurol. Sci 114, 7–12. 10.1016/0022-510x(93)90041-v 8433101

[B40] Scott J. A. Braskie M. N. Tosun D. Thompson P. M. Weiner M. DeCarli C.. (2015). Cerebral amyloid and hypertension are independently associated with white matter lesions in elderly. Front. Aging Neurosci. 7:221. 10.3389/fnagi.2015.00221 26648866 PMC4664630

[B41] Sepulcre J. Goñi J. Masdeu J. C. Bejarano B. Vélez de Mendizábal N. Toledo J. B.. (2009). Contribution of white matter lesions to gray matter atrophy in multiple sclerosis: evidence from voxel-based analysis of T1 lesions in the visual pathway. Arch. Neurol. 66, 173–179. 10.1001/archneurol.2008.562 19204153

[B42] Smith E. E. Salat D. H. Jeng J. McCreary C. R. Fischl B. Schmahmann J. D.. (2011). Correlations between MRI white matter lesion location and executive function and episodic memory. Neurology 76, 1492–1499. 10.1212/WNL.0b013e318217e7c8 21518999 PMC3087468

[B43] Spilt A. Goekoop R. Westendorp R. G. J. Blauw G. J. de Craen A. J. M. van Buchem M. A. (2006). Not all age-related white matter hyperintensities are the same: a magnetization transfer imaging study. Am. J. Neuroradiol. 27, 1964–1968. 17032876 PMC7977888

[B44] Stenset V. Johnsen L. Kocot D. Negaard A. Skinningsrud A. Gulbrandsen P.. (2006). Associations between white matter lesions, cerebrovascular risk factors and low CSF Aβ42. Neurology 67, 830–833. 10.1212/01.WNL.0000234030.77831.5a 16966546

[B45] Swartz R. H. Sahlas D. J. Black S. E. (2003). Strategic involvement of cholinergic pathways and executive dysfunction: does location of white matter signal hyperintensities matter? J. Stroke Cerebrovasc. Dis. 12, 29–36. 10.1053/jscd.2003.5 17903901

[B46] von Bohlen und Halbach O. Unsicker K. (2002). Morphological alterations in the amygdala and hippocampus of mice during ageing. Eur. J. Neurosci. 16, 2434–2440. 10.1046/j.1460-9568.2002.02405.x 12492438

[B47] Waldemar G. Christiansen P. Larsson H. B. Høgh P. Laursen H. Lassen N. A.. (1994). White matter magnetic resonance hyperintensities in dementia of the Alzheimer type: morphological and regional cerebral blood flow correlates. J. Neurol. Neurosurg. Psychiatry 57, 1458–1465. 10.1136/jnnp.57.12.1458 7798973 PMC1073224

[B48] Yamawaki M. Wada-Isoe K. Yamamoto M. Nakashita S. Uemura Y. Takahashi Y.. (2015). Association of cerebral white matter lesions with cognitive function and mood in Japanese elderly people: a population-based study. Brain Behav. 5:e00315. 10.1002/brb3.315 25798332 PMC4356848

[B49] Yoshita M. Fletcher E. Harvey D. Ortega M. Martinez O. Mungas D. M.. (2006). Extent and distribution of white matter hyperintensities in normal aging, MCI and AD. Neurology 67, 2192–2198. 10.1212/01.WNL.0000249119.95747.1f 17190943 PMC3776588

[B50] Zhang Y. Schuff N. Camacho M. Chao L. L. Fletcher T. P. Yaffe K.. (2013). MRI markers for mild cognitive impairment: comparisons between white matter integrity and gray matter volume measurements. PLoS One 8:e66367. 10.1371/journal.pone.0066367 23762488 PMC3675142

